# Complete Genome Sequence of Sequevar 14M *Ralstonia solanacearum* Strain HA4-1 Reveals Novel Type III Effectors Acquired Through Horizontal Gene Transfer

**DOI:** 10.3389/fmicb.2019.01893

**Published:** 2019-08-14

**Authors:** Xiaodan Tan, Huishan Qiu, Feng Li, Dong Cheng, Xueao Zheng, Bingsen Wang, Mengshu Huang, Wenhao Li, Yanping Li, Kangqi Sang, Botao Song, Juan Du, Huilan Chen, Conghua Xie

**Affiliations:** ^1^Key Laboratory of Potato Biology and Biotechnology, Ministry of Agriculture and Rural Affairs, Huazhong Agricultural University, Wuhan, China; ^2^Key Laboratory of Horticultural Plant Biology, Ministry of Education, Huazhong Agricultural University, Wuhan, China; ^3^National Center for Vegetable Improvement (Central China), Wuhan, China

**Keywords:** *Ralstonia solanacearum*, genome sequencing, plasmid, type III effectors, potato

## Abstract

*Ralstonia solanacearum*, which causes bacterial wilt in a broad range of plants, is considered a “species complex” due to its significant genetic diversity. Recently, we have isolated a new *R. solanacearum* strain HA4-1 from Hong’an county in Hubei province of China and identified it being phylotype I, sequevar 14M (phylotype I-14M). Interestingly, we found that it can cause various disease symptoms among different potato genotypes and display different pathogenic behavior compared to a phylogenetically related strain, GMI1000. To dissect the pathogenic mechanisms of HA4-1, we sequenced its whole genome by combined sequencing technologies including Illumina HiSeq2000, PacBio RS II, and BAC-end sequencing. Genome assembly results revealed the presence of a conventional chromosome, a megaplasmid as well as a 143 kb plasmid in HA4-1. Comparative genome analysis between HA4-1 and GMI1000 shows high conservation of the general virulence factors such as secretion systems, motility, exopolysaccharides (EPS), and key regulatory factors, but significant variation in the repertoire and structure of type III effectors, which could be the determinants of their differential pathogenesis in certain potato species or genotypes. We have identified two novel type III effectors that were probably acquired through horizontal gene transfer (HGT). These novel *R. solanacearum* effectors display homology to several YopJ and XopAC family members. We named them as RipBR and RipBS. Notably, the copy of RipBR on the plasmid is a pseudogene, while the other on the megaplasmid is normal. For RipBS, there are three copies located in the megaplasmid and plasmid, respectively. Our results have not only enriched the genome information on *R. solanacearum* species complex by sequencing the first sequevar 14M strain and the largest plasmid reported in *R. solanacearum* to date but also revealed the variation in the repertoire of type III effectors. This will greatly contribute to the future studies on the pathogenic evolution, host adaptation, and interaction between *R. solanacearum* and potato.

## Introduction

*Ralstonia solanacearum*, a soil-borne vascular pathogen that causes bacterial wilt, is a serious threat to crop production worldwide (Hayward, 1991; Genin and Denny, 2012; Mansfield et al., 2012). It is widely distributed throughout the world from tropical and subtropical regions to temperate and cool regions, possibly due to the global climate warming (Elphinstone et al., 2005; Jiang et al., 2017). Its multitudinous and increasing hosts incorporate quite a broad range of plant species, including both monocot and dicot, herbaceous and woody plants, such as tomato, potato, eggplant, peanut, ginger, banana, olive, mulberry, and geranium (Jiang et al., 2017; Bocsanczy et al., 2019). Thus, *R. solanacearum* is a major constraint to the production of several economically important agricultural crops and ornamental plants. Among the species infected by bacterial wilt, *Solanaceous* plants are the most seriously affected. Potato (*Solanum tuberosum* L.), ranked as the third most important food crop, feeds more than a billion people worldwide. However, bacterial wilt affects more than 1.5 million hectares of potato crops with an estimated production loss of more than $950 million per annum (Elphinstone et al., 2005; Patil et al., 2012).

*Ralstonia solanacearum* is composed of multiple genetic groups, therefore, it is considered as a species complex (Fegan and Prior, 2005). In the past decades, several schemes of classification were presented including races, biovars, phylotypes and sequevars according to different criteria (Fegan and Prior, 2005). Recently, Safni et al. (2014) proposed amending the classification into three genospecies on the basis of genome analysis, which comprise of *R. solanacearum* (phylotype II only), *Ralstonia syzygii* (phylotype IV only) and *Ralstonia pseudosolanacearum* (phylotypes I and III). Of all the phylotypes, the phylotype I infects the widest range of plant species. *R. solanacearum* strains isolated from potato composed of all the four phylotypes (phylotype I–IV) strains according to the literature. But the dominant strain is different in different regions. For example, phylotype I and phylotype IV strains are dominant in Japan and Korea and phylotype IIB-1 strains are dominant in China (Xu et al., 2009; Cellier and Prior, 2010; Sagar et al., 2013; Wang et al., 2017).

*Ralstonia solanacearum* enters host roots and invades the vasculature rapidly. As generally acknowledged, the colonized bacteria release copious amounts of exopolysaccharide (EPS) that can block the xylem vessels and prevent water flow. Consequently, plants exhibit wilting symptoms and finally die (Genin, 2010; Genin and Denny, 2012). In addition to EPS, *R. solanacearum* possesses many other general virulence factors including various secretion systems and associated effectors, type IV pili, phytohormones and genes that can help to overcome various stressful environments and compounds (Genin and Denny, 2012). Among the numerous virulence factors, type III effectors are crucial virulence factors and have been extensively studied in recent years (Genin and Denny, 2012). For pathogenesis, type III effectors alter a variety of cellular and physiological processes through intimate associations with plant protein and/or DNA. Some of these effectors are also recognized by plant proteins and modulate host innate immunity as a result of host adaptation to the pathogen (Poueymiro et al., 2009; Feng and Zhou, 2012; Peeters et al., 2013).

The genome sequences are very useful resources to understand plant–pathogen interaction mechanisms and phylogenetic relations between different pathogen species. Thus, to develop strategies to control *R. solanacearum*, more and more strains have been sequenced since the first publication of the GMI1000 genome sequence (Salanoubat et al., 2002). Till now, the genome sequences of 145 strains are available in three public databases (NCBI^1^, Ralsto T3E^2^, and *R. solanacearum* sp.^3^), of which at least 44 genomes have been sequenced with recent PacBio platform or very deep coverage allowing for complete assemblies (Sabbagh et al., 2019). These genome data play an irreplaceable role in analyzing the genomic diversity and evolution within the *R. solanacearum* species complex, studying the genes contributing to host-range and characterizing the global regulation mechanisms that govern the bacterial virulence (Genin and Denny, 2012). Although plethora of genomes have been sequenced, more genome sequences are still needed to analyze the entire species, since new pathotypes are continuously isolated and identified (Bocsanczy et al., 2019). In addition, comparative genomic analysis has been widely used to identify genes contributing to virulence/hypovirulence or host specificity of a particular pathogen such as *R. solanacearum* (Gonzalez et al., 2011; Jalan et al., 2011, 2013; Siri et al., 2014).

Recently, our group has identified a *R. solanacearum* strain HA4-1, also addressed as PeaHuB4, to be phylotype I, sequevar 14M and biovar 3 (Wang et al., 2017). HA4-1 is shown to be highly virulent in most given potato species, while hypovirulent, even avirulent in other potato species. Notably, HA4-1 showed contrasting virulence in certain potato species when compared with GMI1000 and UW551, the latter being the major pathovars infecting potato in most regions (Hayward, 1991; Fegan and Prior, 2005; Xu et al., 2009; Janse, 2010; Wang et al., 2017). To dissect the differential pathogenesis of HA4-1 and other strains, we sequenced and analyzed the HA4-1 genome by combined strategies including Illumina HiSeq2000, PacBio RS II platform and genomic BAC-end sequencing. Then we performed an exhaustive comparative genome analysis specifically focused on the virulence factors.

## Materials and Methods

### Preparation of Strains

The *R. solanacearum* strain HA4-1 was isolated from Hong’an county in Hubei province of China and was identified as phylotype I, sequevar 14M, biovar 3 (Wang et al., 2017). Glycerol stock of *R. solanacearum* was revived from ultra-cold storage freezer by streaking on BGT solid medium [10 g/L peptone, 2.5 g/L glucose, 1 g/L casamino acids, 50 mg/L triphenyltetrazolium chloride (TTC), 15 g/L agar] under sterile conditions and incubated at 28°C for 48–72 h. The pearly cream-white, flat, irregular, fluidal colonies displaying characteristic pink in the center are typical *R. solanacearum* clones. Then typical *R. solanacearum* clones were streaked on new BGT medium plates and incubated at 28°C for 48 h. These clones were further inoculated into 1.5 ml microfuge tubes containing BG liquid medium [BGT medium without TTC and agar, (Boucher et al., 1985)] and grown overnight at 28°C with shaking at 200 rpm. The purity of bacteria was roughly checked under the microscope before proceeding to the subsequent experiments.

### Inoculation Assay

The potato seedlings were inoculated in Murashige-Skoog (MS) medium (supplemented with 4% sucrose and 0.75% agar) and incubated for approximately 3 weeks *in vitro* at (20 ± 1)°C with a 16 h/8 h (day/night) regime. The seedlings were then transferred into pots containing nutritional soil and grew in a greenhouse at (22 ± 2)°C and 75% humidity. After 3–4 weeks, the seedlings having 5–7 full-grown leaves were transferred to inoculation room and incubated at 28°C. After 2 days of adaption to the higher temperature, the plants were inoculated with *R. solanacearum*. The freshly prepared 20–50 μl of bacterial suspension culture was added into a conical flask with BG liquid medium and grew overnight to an OD_600_ of approximately 1.0. Then, it was centrifuged at 4000 rpm for 15–20 min. The pellet was resuspended in water and adjusted to OD_600_ of 0.1 (≈1 × 10^8^ CFU/ml). For inoculation assay, 10 ml of prepared bacterial suspension was added into the soil matrix around the roots, which were freshly cut from the side by a sterile knife. More than six plants from each potato line were inoculated. The disease score of each plant was then recorded every 2 days consecutively for 3 weeks after infection, using the following scales: 0 = plants without visible symptoms; 1 = 1–25% of leaves wilted; 2 = 26–50% of leaves wilted; 3 = 51–75% of leaves wilted; 4 = 76–100% of leaves wilted. This experiment was repeated in triplicates. The degree of the disease was evaluated using the Disease Index (DI) (DI = Σ n*i*/N, where, *i* is the disease score of the plants; n is the number of plants showing disease score of *i*; N is the total number of the plants inoculated). All the experiments were performed according to the experiment security regulations of Huazhong Agricultural University (HZAU), and approved by the biosafety committee in HZAU.

### Morphological Observation and Growth Rate Measurement of the Strains

The morphology of the bacterial clone was determined using suspension culture in the exponential phase, which was diluted, spread onto the TTC medium, and then incubated at 28°C. To measure the growth rate, 100 μl of bacterial suspension of OD_600_ = 0.5 was added to 50 ml of BG liquid medium in a conical flask and cultured under shaking condition at 200 rpm at 28°C. Every 2 h, 1 ml of the growing culture was pipetted out to measure the absorbance value at 600 nm. This experiment was repeated thrice.

### Genome Sequencing, Assembly, and Gap Closure

The genomic DNA of HA4-1 strain was purified from overnight grown liquid cultures using Blood & Cell Culture DNA Mini Kit (Qiagen) by following manufacturer’s instructions for Gram-negative bacteria. The genome sequencing was performed by combining different strategies. Firstly, a paired-end library with an insert size of 500 bp was sequenced using an Illumina HiSeq2000 using PE100 strategy. Genome assembly was performed using Velvet (1.2.07). Then, the single molecule real-time (SMRT) sequencing was performed on the PacBio RS II platform with a 20 kb library. Sequencing reads were assembled *de novo* by following the Hierarchical Genome Assembly Process (HGAP) workflow (PacBioDevNet; Pacific Biosciences) available in SMRT analysis version 2.3.1 (Chin et al., 2013). For gap closure and assembly correction, a BAC library containing 1536 clones was constructed with an average insert fragment of 94 kb and the clones were validated by BAC-end sequencing and PCR.

### Genome Annotation

The coding sequences were predicted using Prodigal (Hyatt et al., 2010). Circular maps of the genome were drawn by CGview (Grant and Stothard, 2008). The genome sequence of HA4-1 was aligned with GMI1000 and UW551 using MuMmer program (Chen et al., 2006). Functional annotation analysis was based on the NCBI protein databases Nr (non-redundant), KEGG, Pfam, Swissprot, COG and nucleotide database Nt. Cluster of Orthologous Group of proteins (COG) was analyzed to determine the functional annotations of the CDS (reference to orthologous groups) (Tatusov et al., 2001). The GIs in the HA4-1 genome were predicted using three methods, namely, IslandPath-DIMO, SIGI-HMM, and IslandPick of Island Viewer (Dhillon et al., 2015). PHAST (PHAge Search Tool) was used to predict the bacteriophage sequences (Tatusov et al., 2001).

### Sequence Analysis

Homology alignment of the full coding DNA sequences (CDSs) and protein sequences of the compared pairs between HA4-1 and GMI1000 were performed by Basic Local Alignment Search Tool (BLAST) of NCBI. For phylogenetic analysis, protein sequences of YopJ effector family were obtained from the NCBI according to the literature (Ma and Ma, 2016). The YopJ family members of *R. solanacearum* were obtained from Ralsto T3E database. The phylogenetic analysis was inferred using the Neighbor-Joining method (Saitou and Nei, 1987). The evolutionary distances were computed using the Poisson correction method (Zuckerkandl and Pauling, 1965). Evolutionary analyses were conducted in MEGA X (Kumar et al., 2018). The sequence alignment was performed using MEGA X and JavaView.

### Statistical Analysis

Statistical analysis was performed by GraphPad Prism 7.

## Results

### HA4-1 Strain Shows Peculiar Pathogenesis in Potato Compared to Other *Ralstonia solanacearum* Strains

**FIGURE 1:**
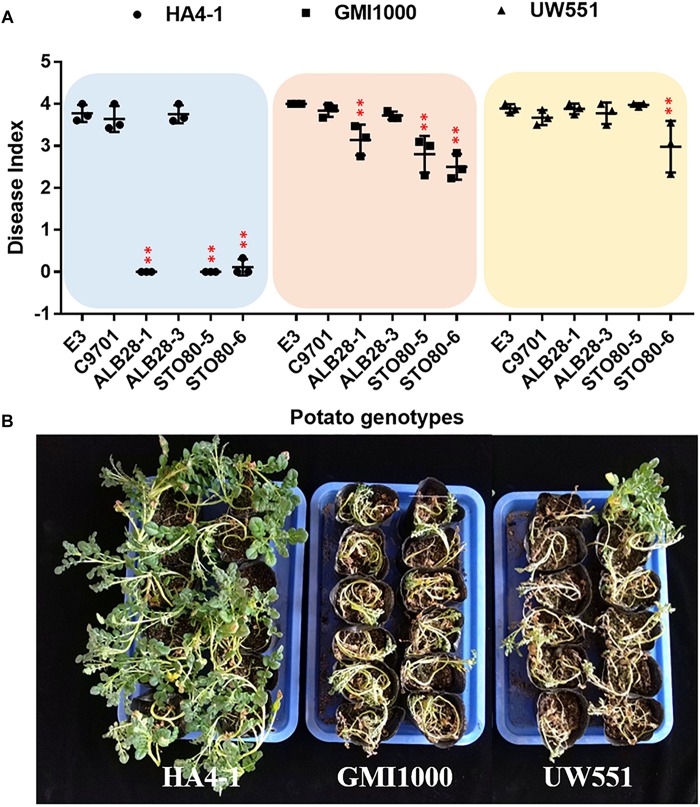
Pathogenicity of three *Ralstonia solanacearum* strains in potato. **(A)** Pathogenicity characterization of *R. solanacearum* HA4-1, GMI1000, and UW551 to six different potato genotypes (*S. tuberosum* cv. E3, *S. chacoense* acc. C9701, *S. albicans* acc. ALB28-1 and ALB28-3, *S. stoloniferum* acc. STO80-5 and STO80-6). The disease index was calculated using the data collected at 14 days post inoculation (dpi) (mean ± SD, *n* = 3, ^∗∗^*P* < 0.01, ^*^P < 0.05, Dunnett-test). Asterisks indicate significant differences compared to *S. tuberosum* cv. E3. **(B)** A representative showing of different symptoms caused by different pathogen strains on the same genotype (ALB28-1). Pictures were taken at 14 dpi.

Previously, the pathogenicity of *R. solanacearum* strain HA4-1 was tested in different potato species. HA4-1 showed varied degrees of virulence in different genotypes. In this study, six potato genotypes from four species (Supplementary Table S1) were selected to compare its virulence with two other strains, GMI1000 and UW551. Consistent with previous results, three genotypes named *S. tuberosum* cv. E-potato 3 (E3), *Solanum chacoense* acc. C9701 and *Solanum albicans* acc. ALB28-3 were susceptible to HA4-1, which could kill most of the plants tested in 10–14 days. In contrast, three other genotypes named *S. albicans* acc. ALB28-1, *Solanum stoloniferum* acc. STO80-5 and STO80-6 were extremely resistant to HA4-1. For GMI1000, all six tested genotypes were moderate susceptible or high susceptible. While for UW551, they were even more susceptible (Figure 1). These results indicate that HA4-1 has a limited pathogenic spectrum compared to GMI1000 and UW551. Notably, we also found that different genotypes of the same accession (e.g., ALB28) showed significantly different disease symptoms when inoculated with HA4-1, as ALB28-1 was highly resistant while ALB28-3 was highly susceptible. Disease index trend during 14 days and comparison at 14 dpi of each potato genotype after soil soak inoculated by three strains were shown in Supplementary Figure S1.

### Genome Sequencing and Assembly Reveal a Small Plasmid in HA4-1

To understand the interaction mechanism of HA4-1 with potato from the pathogen aspect, Illumina HiSeq2000 and single molecule real-time sequencing (SMRT) on the PacBio RS II platform were successively used to sequence the genome of HA4-1. Clean data of 2395 Mb and 500 Mb were generated, respectively. The clean data from Illumina HiSeq2000 were assembled into 423 contigs, and those from the PacBio RS II platform were assembled into three scaffolds of about 5.84 Mb, 87 kb, and 44 kb, respectively. The 423 contigs could be perfectly mapped onto the three scaffolds. However, the genetic material of *R. solanacearum* conventionally comprises of bipartite genome that includes two circular replicons (Genin and Denny, 2012), namely, a chromosome (about 3.8 Mb) and a megaplasmid (about 2.0 Mb), although some strains have small plasmids as well (Morales and Sequeira, 1985; Remenant et al., 2010; Ailloud et al., 2015; Cho et al., 2019). To rectify the assembly, the three scaffolds were realigned with the genome sequences of *R. solanacearum* GMI1000 and four long repeat regions of rRNA in the HA4-1 genome were found located on the 5.84 Mb and 87 kb scaffolds. The incorrect assembly in the rRNA regions was analyzed by the genes flanking these regions. The assembly results were corrected using GMI1000 genome as a reference and further confirmed by long PCR sequencing. Finally, the HA4-1 genome was reassembled into a circular chromosome of about 3.8 Mb and a megaplasmid of about 2.0 Mb with a gap.

**FIGURE 2:**
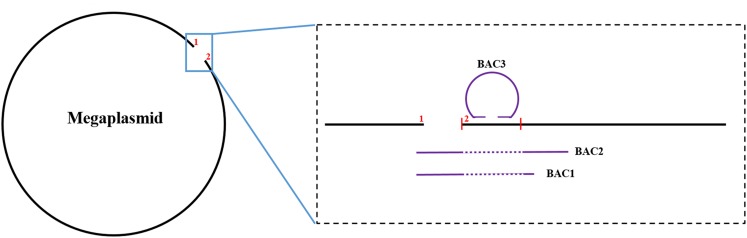
The illustration of the gap closure of the megaplasmid with BAC sequences. The black line and the purple line represent the megaplasmid sequence and the BAC sequence, respectively. The region between the two short red vertical lines belongs to the small plasmid. “1” and “2” indicate the positions of the two gap borders. The dotted portions of the purple line indicate absence of the region in BAC1 and BAC2.

To close the gap on the megaplasmid, we screened a few BAC clones of the HA4-1 genome by PCR and obtained three targeted BACs. BAC1 and BAC2 both screened by the marker at the gap border 1 stretched across the gap with BAC2 being longer than BAC1 (Figure 2). BAC3 was screened by the marker at gap border 2. After end-sequencing of the BAC2 and BAC3, we surprisingly found both the ends of BAC3 at the gap border 2 region in an end-to-end status, which suggested that the gap border 2 region may belong to a small plasmid. Moreover, this putative plasmid region was absent in BAC2 as verified by PCR subsequently (Figure 2). Finally, we found that the gap border 2 region of the megaplasmid scaffold is actually a small plasmid by intensive PCR sequencing. After excising the sequence belonging to the small plasmid from the megaplasmid, a new gap border 2 of the megaplasmid was generated. PCR product stretching across the gap region was obtained with primers at the gap border 1 and the new gap border 2. Sequencing results showed that the gap was about 4 kb. To analyze the 44 kb scaffold, we fortunately found two overlapping BACs that covered the whole region and spread to the chromosome on both sides, which suggested that the scaffold belonged to the chromosome. Thus, the HA4-1 genome comprises of a circular chromosome and a circular megaplasmid with no gaps. In addition, we also identified a small plasmid which was named as pRSHA.

### General Features of the HA4-1 Genome

**FIGURE 3:**
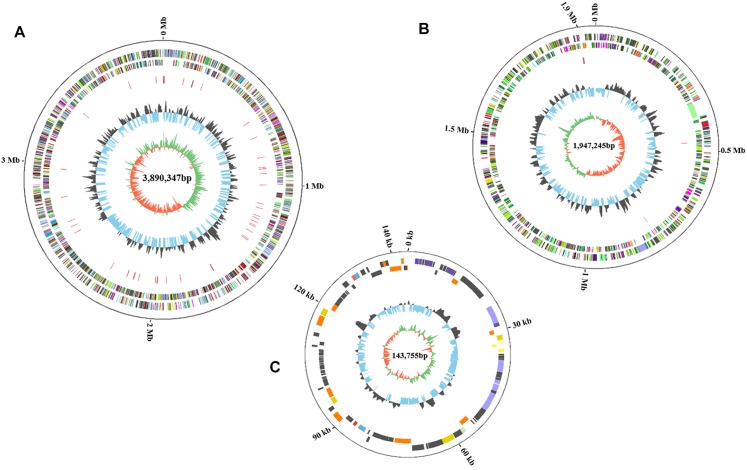
The circular maps of the HA4-1 genome chromosome **(A)**, megaplasmid **(B)**, and small plasmid **(C)**. The size of chromosome is 3,890,347 bp, megaplasmid is 1,947,245 bp and small plasmid is 143,755 bp. The circles from outer to inner represent the genome size, forward CDS and reverse CDS (different colors represent different functional classifications), rRNA and tRNA (blue, rRNA; red, tRNA), GC ratio (black, outward means GC ratio of the region is higher than average GC ratio; blue, inward means GC ratio of the region is lower than average GC ratio), GC skew (green represents a region with G content greater than C, orange represents a region with C content greater than G).

**FIGURE 4:**
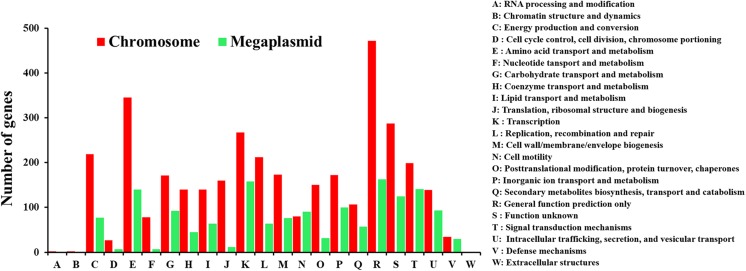
The distribution of genes with COG functional categories in the chromosome and the megaplasmid of HA4-1.

The complete genome of HA4-1 is 5.84 Mb (GC%, 66.68%), which comprises a 3.89 Mb circular chromosome (Figure 3A) and a 1.95 Mb circular megaplasmid (Figure 3B). The general features of the HA4-1 genome are listed in Table 1. In total, 4,930 CDSs have been predicted with 3,439 in the chromosome and 1,491 in the megaplasmid. The HA4-1 genome contains 12 rRNAs and 59 tRNAs. Functional annotation successfully classified 3791 genes into 23 COG categories, among which, categories of cell motility and defense mechanisms harbor similar amount of genes in chromosome and megaplasmid (Figure 4). In total, 56 genomic islands (GIs, Supplementary Figure S2 and Supplementary Table S2) and nine prophages (Supplementary Figure S2 and Supplementary Table S3) have been predicted, which accounts for 49 and 12% of the HA4-1 genome, respectively. In addition, there is a plethora of integrase and transposase genes (1.93%) in the HA4-1 genome.

**TABLE 1:** Genomic features of the *Ralstonia solanacearum* stain HA4-1.

**Feature**	**Value**
Genome size (bp)	5,837,592
Chromosome (bp)	3,890,347
Megaplasmid (bp)	1,947,245
Plasmid (bp)	143,755
GC content (%)	66.68
Predicted CDSs	4,930
rRNA	12
tRNA	59
Genomic islands	54
Prophage regions	9

### The Plasmid pRSHA Carries Type IV Secretion System and Type III Effectors

**FIGURE 5:**
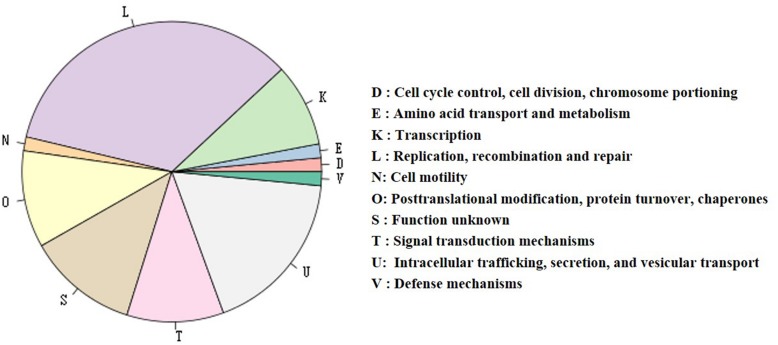
Distribution of genes with COG functional categories of plasmid pRSHA. The gene numbers of each category are as follows (from most to less): 23 genes in L category, 12 in U, 8 in S, 7 in O, 7 in T, 6 in K, 1 in each category of D, E, N, and V. The Vir-type T4SS gene cluster is in the U category. RipBS is in S category. RipBR is not classified into any categories.

The plasmid pRSHA (Figure 3C), 143,755 bp in size, is the largest plasmid among the five presently sequenced in *R. solanacearum* strains (Supplementary Table S4). It is widely syntenic with pRST78 and pUW163a/b (Supplementary Figure S3), while shares no synteny with others. In total, the plasmid was predicted to contain 123 CDSs, a genome-island and an incomplete prophage. 50 genes of the plasmid were successfully classified into 10 COG functional categories (Figure 5), among which the largest group of genes (18 genes) are involved in replication, recombination and repair, followed by 12 genes involved in intracellular trafficking, secretion, and vesicular transport. The 12 genes were identified to encode a Vir-type type IV secretion system, including nine genes for the virB operon (virB3, 4, 5, 6, 8, 9, 10, and 11) and virD4 (Supplementary Figure S4). In addition, the region from position 133,918 bp to 143,519 bp comprising transposable elements flanked by short repeats was identified to belong to Tn3 transposon family. Four genes (CFM90_26590, CFM90_26600, CFM90_26510, and CFM90_26330) were annotated as type III effectors. Further analysis showed that CFM90_26590 and CFM90_26600 genes are actually one gene split by a transposable element insertion. It is a homolog of YopJ family effector genes. CFM90_26510 and CFM90_26330 are two copies and homologs of a type III effector XopAC of *Xanthomonas campestris* pv. campestris (*Xcc*). In addition, we detected standard *hrp*II box (TTCGn16TTCG), which is an imperfect plant inducible promoter (PIP) box (Cunnac et al., 2004a), in the promoters of the two putative type III effectors. Based on the guidelines of the nomenclature of type III effectors in the *R. solanacearum* species complex (Peeters et al., 2013), we named the two effectors RipBR and RipBS, respectively (Table 2).

**TABLE 2:** Distinct type III effector genes between HA4-1 and GMI1000.

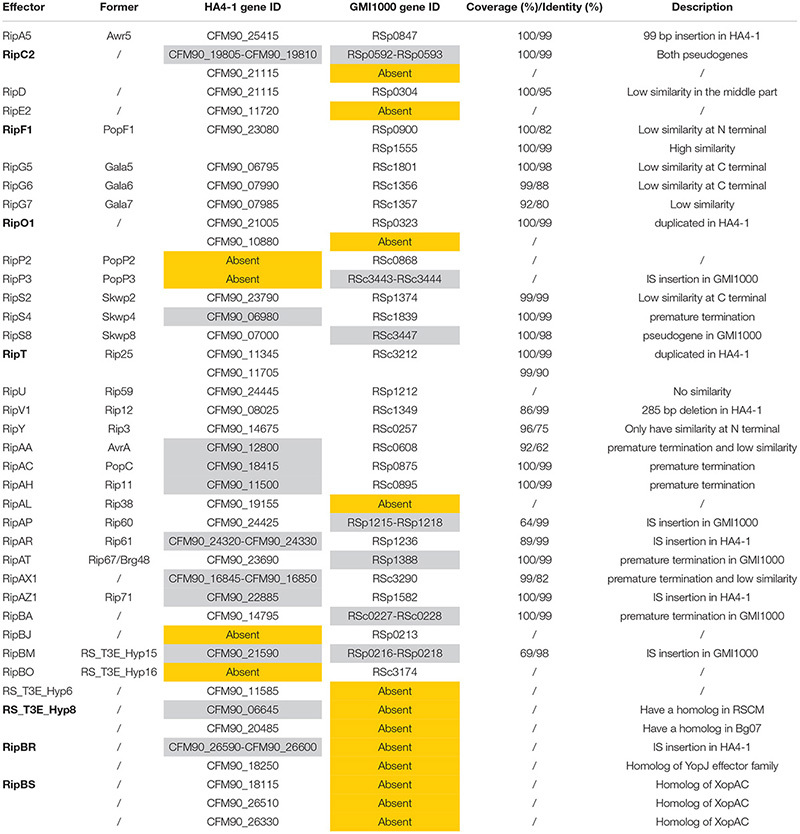

*“Absent” indicates that the effector gene is absent in the corresponding genome. The effector genes in gray table cells are pseudogenes. Table cells with diagonal have no data. The bold effector genes are duplicated in at least one genome.*

### Identification and Comparative Analysis of the Virulence Factors of HA4-1

To gain a deeper insight into strain HA4-1, we compared its morphological characteristics, growth rate, and genome sequence with two other published strains GMI1000 (Salanoubat et al., 2002) and UW551 (Gabriel et al., 2006). The proliferation rate of HA4-1 is higher than that of the other two strains on both solid and liquid medium (Supplementary Figure S5). Generally, genome synteny is higher between HA4-1 and GMI1000 than between HA4-1 and UW551. In total, 4454 (88.1%) CDSs share widespread synteny and collinearity between HA4-1 and GMI1000, while 3674 (72.7%) CDSs share synteny between HA4-1 and UW551 (Supplementary Figure S6). Moreover, nucleotide sequences are more polymorphic and proliferation rates more divergent (Supplementary Figure S5) between HA4-1 and UW551 than between HA4-1 and GMI1000. Notably, HA4-1 and GMI1000 both belong to phylotype I and biovar 3 (Wang et al., 2017), but have different virulence levels in certain potato geno- types such as ALB28-1. Thus, we performed a comparative analysis between the two strains and mainly focused on the general virulence factors such as secretion systems, motility, EPS, and key regulatory factors, as well as the repertoire of type III effectors.

#### Secretion Systems and Related Proteins

Secretion systems in the bacteria have evolved to interact with the external environment and contribute to pathogenicity. Gram-negative bacteria encode at least six different secretion systems (type I-VI) that deliver proteins to the extracellular milieu or directly into the cytosol of host cells (Records, 2011). At present, four (type II–IV, VI) secretion systems are most widely studied in *R. solanacearum*. Here we predicted the genes related to the four secretion systems in HA4-1 and compared them with those in GMI1000.

Type II secretion system (T2SS) produces several virulence factors including a consortium of plant cell wall-degrading enzymes (CWDEs) (Genin and Boucher, 2004). Like GMI1000, HA4-1 also possesses three T2SSs. The first one is the orthodox system encoded by 12 genes in the chromosome from 3,774,266 bp to 3,786,571 bp. Another two are unorthodox systems, with one encoded by 11 genes in the chromosome from 723,800 bp to 733,741 bp and the other encoded by 14 genes in the megaplasmid from 971,137 bp to 980,479 bp. Gene clusters in the three T2SSs are highly conserved between HA4-1 and GMI1000.

Type III secretion system (T3SS) can inject type III effectors into plant cells through a syringe-like structure. It is essential for the pathogenicity or the hypersensitive response (HR) in *R. solanacearum* (Valls et al., 2006; Coll and Valls, 2013). T3SS is composed of hypersensitive response and pathogenicity (hrp) gene clusters present in the megaplasmid (Lindgren, 1997; Genin and Boucher, 2004). In the HA4-1 megaplasmid, the hrp gene cluster containing 30 genes located between 67,335 bp and 96,990 bp shares more than 99% similarity with that of GMI1000 (Supplementary Table S5), which indicates that the discrepancy in virulence between HA4-1 and GMI1000 is not likely due to T3SS structural genes.

For type IV secretion system (T4SS), there are 14 homologs between the two strains (Supplementary Figure S4; Salanoubat et al., 2002; Genin and Boucher, 2004), while HA4-1 lacks the homologous genes between trbJ and trbL. The T4SS genes show similar genetic rearrangements. The similarities vary from 63 to 90% with the counterparts in GMI1000. In addition, the small plasmid of HA4-1 carries a virB operon related to T4SS (Supplementary Figure S4).

Type VI secretion system (T6SS) is widespread among Gram-negative bacteria and has versatile roles including virulence (Records, 2011). The T6SS locus spans 45.457 kb region in the megaplasmid of GMI1000 (Leiman et al., 2009), whereas in HA4-1 megaplasmid, it spans 42.826 kb region from 203,091 bp to 245,916 bp. Within this locus, the genes between vgrG1 and vgrG2 are highly conserved, while vgrG1 itself is relatively variable. Eight genes are located between vgrG2 and impA, among which three are identical in GMI1000 including a glycoside hydrolase family 10 protein, while the other five are variable including an IS5/IS1182 family transposase. The 10 genes from impA and vgrG3 are highly conserved. Previous reports defined the core T6SS genes by comparing several *R. solanacearum* strains including all phylotypes (Li et al., 2016, 2018), among which vasK located between impA and vgrG3 was identified to be important for the pathogenicity in tomato (Zhang et al., 2011). In general, the core T6SS genes in GMI1000 and HA4-1 are highly conserved (Supplementary Table S6).

#### Motility

Gram-negative bacteria produce various types of pili (Fronzes et al., 2014) among which the type IV pili are the most abundant pili described to date. The functions of type IV pili are quite diverse and they were shown to play an important role in adhesion of pathogenic bacteria to their host cells, biofilm formation, twitching motility, conjugative DNA transfer, and bacteriophage infection (Mattick et al., 1996; Wall and Kaiser, 1999; Kang et al., 2002; Craig and Li, 2008; O’Toole and Kolter, 2010). Therefore, it is not surprising that pili are essential virulence factors of many bacterial species (Liu et al., 2001; Tans-Kersten et al., 2001). Similar to GMI1000, there are more than 50 candidate genes for the synthesis and function of the type IV pili in HA4-1 (Supplementary Table S7). Most of the candidate genes are clustered, of which five clusters are located in the chromosome and one in the megaplasmid. Each gene cluster consists of 3–12 genes. In addition to the twitching motility, swimming motility conferred by flagellin is also important to *R. solanacearum*. Comparative analysis shows that all the above-mentioned pili and flagellin gene clusters are highly conserved between HA4-1 and GMI1000 (Supplementary Table S7).

In addition to the type IV pili, some T2SSs are predicted to produce type II pseudopili, a bundled type of pili that increase adhesion capabilities (Genin and Boucher, 2004). Tight adherence (tad) genes might produce another type of bundled pili that mediate adherence and are required for tenacious biofilm formation (Wairuri et al., 2012). Similar to GMI1000, two tad loci have been predicted in HA4-1, which are located in the chromosome and megaplasmid. In addition, the tad locus sequences are extremely similar between the two strains (Supplementary Table S7).

#### Other Virulence Factors

In addition to the above-mentioned genes, many other genes also contribute to the virulence of *R. solanacearum* strains. Thus, we compared these genes as well between HA4-1 and GMI1000. Based on the previous studies, 82 virulence genes excluding the above-mentioned genes were investigated, which include key regulatory factors, EPS biosynthesis genes, elicitins, biofilm and other genes required for infecting host or surviving stressful environments and compounds (Supplementary Table S8). All the analyzed genes are highly conserved with similarity of more than 99%.

### Repertoire and Comparative Analysis of Type III Effectors of HA4-1

According to the Ralsto T3E database (Peeters et al., 2013) and the gene functional annotation, 80 type III effectors were identified in HA4-1, among which 69 are functional genes and 11 are pseudogenes. Comparative analysis of the type III effectors was performed between HA4-1 and GMI1000 (Supplementary Table S9). Ten strain-specific effector genes were identified in HA4-1 and GMI1000. *Rip*P2, *Rip*P3, *Rip*BJ, and *Rip*BO are absent in HA4-1 but present in GMI1000, although *Rip*P3 is a pseudogene. On the contrary, *Rip*E2, *Rip*AL, *Rip*BR, *Rip*BS, *RS_T3E_*Hyp6, *RS_T3E_*Hyp8 are specific to HA4-1. Notably, *Rip*BR has two copies located in the megaplasmid and the small plasmid. The *Rip*BS has three copies, among which two are in the small plasmid and one is in the megaplasmid. Different homologs of *RS_T3E_*Hyp8 were identified in the chromosome and megaplasmid of HA4-1, respectively. One is homologous with the counterpart of *R. solanacearum* RSCM, although they are both pseudogenes. The other one is exactly identical with the counterpart of *R. solanacearum* Bg07. Besides *Rip*BR and *RS_T3E_*Hyp8, three other effector genes (*Rip*C2, *Rip*O1, and *Rip*T) are duplicated in HA4-1. For RipC2, the gene CFM90_21115 has no counterpart in GMI1000, while the other homologs are pseudogenes in both strains. For RipO1, the single copy in GMI1000 share a similarity of 99% with CFM90_21005 of HA4-1, but share no similarity with CFM90_10880 of HA4-1. On the contrary, *Rip*F1 is duplicated in GMI1000 but not HA4-1. Eleven effector genes were identified as pseudogenes in various forms in at least one of the strains, among which *Rip*BM are pseudogenes in both the strains. The CDS of *Rip*BM in HA4-1 was split by a transposon at N terminal, while in GMI1000 was split at the middle. RipAA, RipS4, RipAC, RipAH, and RipAX1 proteins are truncated in HA4-1 due to premature termination of translation, among which RipAA is less similar to the counterpart in GMI1000. *Rip*AR and *Rip*AZ1 have transposon insertion in HA4-1, thus are likely to be non-functional. Similarly, *Rip*AP and *Rip*BA are pseudogenes due to the insertion of transposons in GMI1000. In addition, *Rip*S8 and *Rip*AT are pseudogenes in GMI1000 while their counterparts in HA4-1 are likely to be functional. *Rip*A5 and *Rip*V1 are different in the two strains due to the large insertion or deletion but not due to frameshift. *Rip*A5 harbors a 99 bp repeat insertion while *Rip*V1 harbors a 285 bp deletion in HA4-1. In addition to the genes mentioned above, few other type III effectors show relatively low similarities between the two strains, among which RipU shares very low similarity of 43% with a coverage of 20%. RipD has a 95% similarity but a low identity in the middle portion. RipG7 shares 80% similarity, while RipG5 and RipG6 share similarities below 90% and RipS2 shares 99% similarity with their counterparts but no identities at the C-terminal. RipY shows only 75% similarity at N-terminal and no hits at C-terminal. Besides the above-mentioned differences in type III effectors between the two strains (Table 2), the remaining 49 effectors are conserved with insertions or deletions (indels) ranging from 3 bp to 36 bp without frameshift or few non-synonymous single-nucleotide polymorphisms (NS-SNPs). Thus, our comparative analysis shows that type III effectors are largely variable between the two strains.

**FIGURE 6:**
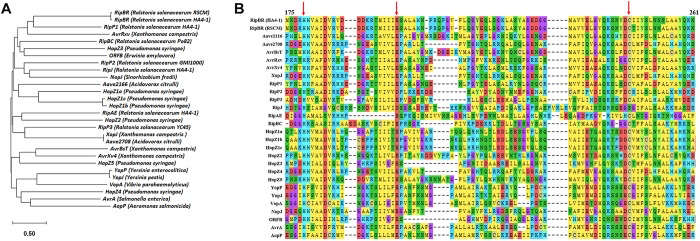
Analysis of RipBR effectors. **(A)** Phylogenetic analysis of known and putative YopJ family effectors. **(B)** Sequence alignment of the RipBR effector of *R. solanacearum* RSCM and HA4-1 with the known and putative YopJ family effectors (Amino acids from 175 to 261 are shown). The red arrows point out the conserved catalytic triad consisting of His/Glu/Cys key residues required for auto-/*trans-*acetylation. The GeneBank accession numbers of effectors used for this analysis are as follows: AKN09807 (YopJ), AAN37537 (YopP), AAL21745 (AvrA), AAT08443 (VopA), NP_710166 (AopP), AAG39033 (AvrXv4), AAA27595 (AvrRxv), AAD39255 (AvrBsT), ABM32744 (Aave2166), ABM33278 (Aave2708), WP_011347382 (XopJ), CAD13849 for AAR02168 (HopZ1a), WP_004661226 (HopZ1b), AAL84243 (HopZ1c), CAC16700 (HopZ2), AAF71492 (HopZ3), EKG29639 (HopZ4), AAF63400 (ORFB), and NP_443964 (NopJ). The effectors of *R. solanacearum* are from the Ralsto T3E database. RipP2 is from GMI1000, RipP3 is from YC45, and RipBC is from Po82. Sequence of RipBR of *R. solanacearum* RSCM is from NCBI. The effectors *R. solanacearum* effectors of HA4-1 are from this study. Phylogenetic analysis and sequence alignment were performed by MEGA X.

**FIGURE 7:**
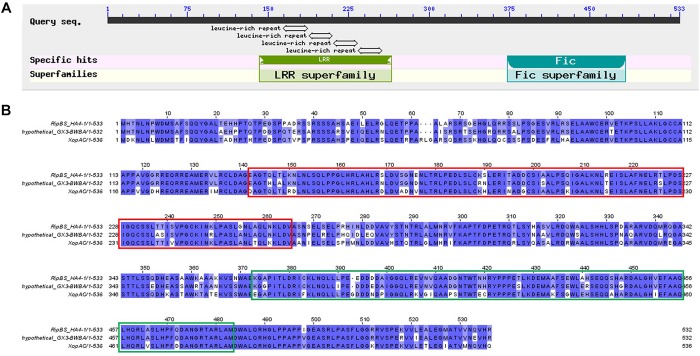
Analysis of RipBS effector. **(A)** Conserved domains of the RipBS effector predicted in NCBI. The LRR domain spans from 142 to 265 and the Fic domain spans from position 373 to 483. **(B)** Sequence alignment of the RipBS effector of *R. solanacearum* HA4-1 with its homologs. Sequences in the red frame is the LRR domain and in the green frame is Fic domain. The GeneBank accession numbers of effectors used for this analysis are as follows: WP_112188676 for the hypothetical protein of *Ralstonia* sp. GX3-BWBA, AFP74845 for the XopAC of *Xanthomonas campestris* pv. campestris str. 8004. Sequence alignment was performed by JavaView.

Then we performed BLAST analysis of the six type III effectors present in HA4-1 and absent in GMI1000 with all the sequenced *R. solanacearum* strains in the NCBI database. Most strains have RipE2 and RipAL homologs with high similarities. For RipBR, only RSCM (PRJNA422474^4^) has a homolog on the megaplasmid, which shares 96% similarity at the nucleotide level with the megaplasmid-born copy in HA4-1 and is identical with the plasmid-borne copy when not considering the transposon. BLASTP analysis shows that RipBR shares high similarity with several YopJ family effectors including AvrBsT, AvrRxv of *X. campestris*, Aave2166 and Aave2708 of *Acidovorax citrulli* and HopZ5 of *Pseudomonas syringae*. Therefore, phylogenetic analysis of known and putative YopJ family effectors was performed. Result shows that RipBR is closest to a known type III effectors of *R. solanacearum* PopP1 (RipP1) (Figure 6). For RipBS, no homologs have been identified in any of the sequenced *R. solanacearum* strains at the nucleotide level, however, further BLAST analysis revealed a homolog in *Xcc* with 100% coverage and 85% similarity. At the amino acid level, another homolog in *Ralstonia* sp. GX3-BWBA was identified with 99% coverage and 88% similarity (Figure 7). The known type III effector RipAC (PopC) of *R. solanacearum* shares less than 35% similarity with RipBS. Thus, RipBS was first identified in *R. solanacearum*. For the indel-harboring effectors RipA5 and RipV1, no similar indels were found in the homologs in other strains, which indicates that the indels specifically occurred in strain HA4-1.

## Discussion

As a complex pathogen species, *R. solanacearum* has a broad range of hosts. It has evolved rapidly and can affect diverse hosts worldwide (Genin, 2010; Genin and Denny, 2012). The genomic plasticity imparts diverse pathogenic behavior to *R. solanacearum*. In our study, we identified the contrasting virulence of HA4-1 in certain potato species and then made extensive efforts to elucidate the complete genome sequences of HA4-1. Comparative analysis between HA4-1 and GMI1000 revealed the diverse repertoire of type III effectors, which may be relevant to their contrasting pathogenic behaviors. Our study reports for the first time the genome of the newly identified sequevar 14M *R. solanacearum* strain. The results will not only enhance the information of the *R. solanacearum* genome but also contribute to the identification of variable and novel type III effectors in order to gain a deeper insight into the pathogenic evolution of *R. solanacearum*.

Previously, several genomic comparison studies were performed in order to identify specific differences in gene content of *R. solanacearum* corresponding to low or high virulence in different hosts. A deletion of 33.7 kb was found in the megaplasmid of strain UY043 isolated from soil by comparative genomic hybridization analysis on a spotted microarray (Cruz et al., 2014). This region contains a cluster of six genes involved in type IV pili synthesis, which contribute to early bacterial wilt pathogenesis and colonization fitness of potato roots. A similar finding used suppressive subtractive hybridization to compare a potato *R. solanacearum* II B-1 strain with a related strain isolated from a water course (Stevens and van Elsas, 2010). A deletion of a putative genomic island of 17.6 kb PGI-1 was identified in the environmental strain. The PGI-1 does not affect pathogenicity in tomato, but it was proposed to be involved in colonization fitness and adaptation to the plant local conditions (Stevens and van Elsas, 2010; Stevens et al., 2011). Another case of *R. solanacearum* was reported for two sequenced II B-1 strains IPO1609 and UW551. The two strains are closely related but differ significantly in the virulence in their host plants. The research revealed that IPO1609 indeed carries a 77 kb genomic deletion, which is responsible for almost complete loss of pathogenicity of the strain (Gonzalez et al., 2011). Notably, the strains used in all the above-mentioned research studies belong to phylotype II B-1 or biovar 2, without complete genome sequence information. All the determinants revealed in these cases were involved in general virulence factors, indicating the environmental strains might have lost the natural ability to infect their host. According to our study, HA4-1 is highly virulent in most potato genotypes, which indicates that it retains its intrinsic pathogenicity. Therefore, the avirulence of HA4-1 to certain potato genotypes probably owing to host-specificity. Based on the complete genome sequence, we could perform the genome-wide analysis of the potential determinants imparting pathogenicity and hypovirulence to HA4-1 and determine the novel type III effectors.

Although numerous virulence factors contribute to the pathogenicity of *R. solanacearum* strains, the type III effectors are frequently associated with host-specificity and pathogen evolution. In this study, we compared most of the reported and predicted virulence factors and found that the general virulence factors are highly conserved except some type III effectors. The genome-wide comparative analysis of HA4-1 with other strains indicates that the type III effectors could determine host specificity at species and genotype levels. Previous studies also showed similar results. Comparative genomic analysis of *Xanthomonas axonopodis* pv. citrumelo strain F1 with citrus canker pathogen *X. axonopodis* pv. citri strain 306 revealed several effectors that might be responsible for survival and the low virulence of this pathogen in citrus. Unique effectors were also identified in *X. axonopodis* pv. citrumelo, which may be related to the difference in host range (Jalan et al., 2011, 2013). Comparative genomic analysis of the phylogenetically related *R. solanacearum* species with contrasting biological characteristics was performed to study host adaptation and the emergence of ecotypes. Although relatively few differences in gene content were identified, several type III effectors were probably associated with the Moko, NPB and brown rot ecotypes (Ailloud et al., 2015). Recently, Cho et al. (2019) predicted host-specific genes and effectors *in silico* by pan-genome analysis of 25 Korean potato *R. solanacearum* strains (phylotype I and IV), which may be responsible for pathogenicity in different genera of Solanaceae.

Type III secretion system effectors have been known to contribute to pathogenicity and multiplication of pathogens in plants (Gijsegem et al., 1993; Deslandes and Genin, 2014). T3SS effectors benefit the pathogens by suppressing plant defense mechanisms where these virulence factors alter the physiology of the host cell or by narrowing the host range when certain effectors are specifically recognized as avirulence factors by the host (Jones and Dangl, 2006). Compared with other well-known plant pathogens such as *Pseudomonas* spp. or *Xanthomonas* spp., repertoire of *R. solanacearum* type III effectors is unusually larger (Zumaquero et al., 2010; Hajri et al., 2012). Extensive functional redundancy and variability between effectors are frequent in this strain, which contribute to its wide host range and host-specificity (Cunnac et al., 2004b; Macho et al., 2010). In *R. solanacearum*, many effectors have been identified as virulence factors or contributing to bacterial fitness during infection (Macho et al., 2010; Deslandes and Genin, 2014). In addition, a single type III effector gene is able to extend the host range of *R. solanacearum* GMI1000 as shown by GALA7 (RipG7) effector (Wang et al., 2015b). A similar case is seen in *X. axonopodis*, where pthA effector gene from the *X. axonopodis* pv. citri can confer the ability to cause raised pustules on *X. axonopodis* pv. citrumelo (Swarup et al., 1992). On the contrary, presence of some effectors can limit the host range of the strain. For instance, AvrA (RipAA) determines the incompatibility of some *R. solanacearum* strains by eliciting an immune response in *Nicotiana tabacum* (Carney and Denny, 1990; Poueymiro et al., 2009). PopP1 (RipP1) acts as a host specificity determinant specific to tobacco and petunia (Lavie et al., 2002; Poueymiro et al., 2009), whereas PopP2 (RipP2) is responsible for the incompatibility of GMI1000 and *Arabidopsis thaliana* Nd-1 (Deslandes et al., 1998, 2003). Recently, RipAX2 (Rip36) was identified to induce a strong HR response in leaves of a wild relative of the eggplant and trigger resistance in the eggplant accession AG91-25 (Nahar et al., 2014; Morel et al., 2018). In other words, these effectors are likely to be recognized as avirulence factors specifically by certain plants. In fact, PopP2 (RipP2) is a definite avirulence effector recognized by the Arabidopsis RRS1-R resistance protein (Deslandes et al., 2003). In this study, we identified 80 effectors, among which 69 are likely to be functional in HA4-1. Comparative analysis of the effectors between HA4-1 and GMI1000 strains revealed variable repertoire and wide variation in certain effectors, which may define the difference in virulence and host range between the two strains. Furthermore, HA4-1, and not GMI1000, displayed contrasting interactive phenotypes with two genotypes of the same accession ALB28 of *S. albicans*, which indicates that specific effectors may play significant role in the interaction process. To have a better understanding of the biological mechanisms underlying host adaptation, the type III effectors essential for virulence need to be elucidated.

Among the various effectors identified in this study, several effectors are HA4-1 strain-specific. Although RipA5 and RipV1 are present in most *R. solanacearum* strains, the indels of more than 99 bp are special in HA4-1. A previous study has shown that transient overexpression of RipA5 in the non-host tobacco species shows characteristics of a typical HR (Monteiro et al., 2012). Moreover, RipA5 can cause a decrease in the activity of TOR-regulated nitrate reductase in plants to maintain normal levels of TOR and the Cdc55 homologs, required for *R. solanacearum* virulence (Popa et al., 2016). If and how the 99 bp repeat insertion in ripA5 of HA4-1 alters its function needs further investigation. RipV1 was predicted to contain a putative ubiquitin-ligase domain (Peeters et al., 2013). The role of RipV1 in the interaction of *R. solanacearum* and host plants remains to be determined. If the deleted region affects the ubiquitin-ligase activity also needs further investigation. RipBR, the copy on the megaplasmid and the homolog in RSCM, belongs to YopJ effector family, which is a unique family because its members are from both animal and plant pathogens. The plant pathogens include *Pseudomonas*, *Xanthomonas*, *Acidovorax*, and *Ralstonia* (Figure 6A). The HopZ family of effectors are found in many *P. syringae* strains. AvrXv4, AvrRxv, AvrBsT, XopJ are from *X. campestris* pv. vesicatoria, the causal agent of tomato bacterial spot disease. Several known type III effectors of *R. solanacearum* are members of YopJ effector family or contain acetyltransferase domain including RipP1, RipP2, RipJ, RipAE, and RipBC (Peeters et al., 2013; Ma and Ma, 2016). Many YopJ effectors triggers accession-specific HR and/or immunity and modify their target by acetylating specific serine, threonine, and/or lysine residues. Plant resistance against YopJ family members has been genetically characterized for HopZ1a, AvrBsT, and PopP2 so far (Ma and Ma, 2016; Jayaraman et al., 2017). Domain analysis showed that the megaplasmid-born RipBR effector of HA4-1 and the ones in RSCM both contain the conserved catalytic triad consisting of His/Glu/Cys required for auto-/*trans-*acetylation (Figure 6B), which indicates its potential function may be similar with other members of YopJ effector family. The homolog of RipBS in *Xcc* is the type III effector XopAC, which was identified as an avirulence factor recognized in vascular tissues (Wang et al., 2015a). Similar to XopAC (Xu et al., 2008), RipBS also encodes a protein with an N-terminal LRR domain and a C-terminal fic (filamentation-induced by cAMP, consensus HPFxxG/ANGR) domain (Figure 7). XopAC can impart fic-dependent avirulence to *R. solanacearum* and the mesophyll pathogen *P. syringae* in Arabidopsis ecotype Col-0 (Guy et al., 2013). Therefore, there is the possibility that RipBS could be an avirulence effector in *R. solanacearum* to certain potato lines. Before this study, XopAC was deemed to be only present in *Xcc*. This indicates that the RipBS effector might be horizontally transferred from *Xcc.* The high conservation of the RipBS between the two genera indicates its significance in the pathogenicity of phytopathogens. Whether RipBR and RipBS effectors are avirulence factors in the resistant potato genotypes such as *S. albicans* ALB28-1 needs to be studied. If and how the four type III effectors contribute to the different host ranges and pathogenicity in the *R. solanacearum-*potato interaction needs further exploration. At present, whether these effectors are secreted through the type III secretion system of *R. solanacearum in vitro* and *in vivo* needs to be established and confirmed by experiments such as cAMP assays (Mukaihara et al., 2010; Poueymiro et al., 2014).

Horizontal gene transfer (HGT) plays an important role in *R. solanacearum* genetic and pathogenic diversity by contributing to the rapid acquisition of novel adaptive functions, and thus is important for the adaptation and the emergence of pathogenic variants (Genin and Boucher, 2004). Many *R. solanacearum* genomic islands correspond to putative prophages or phage-like elements, since phages are abundant in soil and key factors in shaping evolution of soil microbial communities (Genin and Denny, 2012). Genes associated with HGT and insertion sequence (IS) elements may encode or disrupt functional proteins including type III effectors in *R. solanacearum* and contribute to virulence or differential host ranges of the strain (Peeters et al., 2013). For example, *Pop*P2 (*Rip*P2) is reported as a bacteriophage-borne type III effector gene that might be required through HGT (Lavie et al., 2004; Guidot et al., 2007). In HA4-1, *Rip*E2 and *RS_T3E_*Hyp6 are located in the GI and/or prophage regions, while *Rip*AR, *Rip*AZ and *Rip*BM are disrupted by IS elements. Small plasmid in pathogens may also contribute to efficient HGT and pathogenicity. Type III effectors such as *pth*A gene, a well-known pathogenicity determinant that causes citrus bacterial canker of *Xanthomonas* spp., is located in the pXAC64 plasmid (Da et al., 2002; Al-Saadi et al., 2007). The *Rip*BS located in the plasmid may be horizontally transferred from *X. campestris* pv. *campestris* and play an important role in the pathogenic evolution of HA4-1. Moreover, the low GC content in *Rip*BR and *Rip*BS add the evidence of HGT. In fact, numerous type III effectors of *R. solanacearum* were probably transferred horizontally from *P. syringae* and *Xanthomonas* according to phylogenetic analyses (Peeters et al., 2013). The gene clusters of T4SS located in the pRSHA plasmid may be also important for the HGT, since the T4SS-mediated horizontal transfer of genes between the plasmid and chromosome contributes to the plasticity of the genome, evolution of the pathogenic bacteria, diffusion of the antibiotic resistance and other virulence genes (Genin and Boucher, 2004). Among the sequenced *R. solanacearum* plasmids, pRSC35 also has a T4SS, although the virB operon is not homologous to that located in pRSHA.

There are many repeats and similar regions in the pRSHA plasmid and megaplasmid of HA4-1. For example, *Rip*BS has copies both in the plasmid and megaplasmid. Therefore, it is plausible that content exchange between the plasmid and the megaplasmid occurred. This is consistent with the previous report that there are drastic variations in the megaplasmid (Genin and Denny, 2012). These observations provide evidence that plasmid plays an important role in strain evolution. Naturally transformation competent of most *R. solanacearum* strains in culture, and probably within plants, may play an essential role in the occurrence of HGT, thus contributing to its genomic plasticity and the emergence of new pathotypes (Bertolla et al., 1999; Coupat-Goutaland et al., 2010).

Among the sequenced *R. solanacearum* plasmid, pRSHA and pRST78 share a high similarity of more than 98%, indicating their likely common origin. However, some regions are specific in one of the two plasmids. For example, the RipBR and RipBS are specific in pRSHA. To explain this phenomenon, we propose three hypotheses: (i) Strain HA4-1 and T78 obtained their own small plasmid from the same kind of bacteria at different times. (ii) The two strains obtained the same plasmid from other bacterium and then evolved into different version to adapt to different niches. (iii) The small plasmid in one strain evolved into a new version and then transferred to another strain. The presence of type IV secretory system in chromosome and small plasmid is conducive to such transferring. No similarities between pRSHA and other sequenced *R. solanacearum* plasmid probably indicating their different origin.

Although small plasmids are found not rarely in *R. solanacearum* strain, detailed sequence information is seldom reported (Morales and Sequeira, 1985). Among the numerous sequenced *R. solanacearum* strains, only five strains have yet been confirmed to harbor plasmids (Remenant et al., 2010). There are three probable reasons: (1) the presence of the small plasmids is the exception rather than the rule; (2) genome assemblies of most sequenced *R. solanacearum* strains are in “draft” status; (3) the plasmids are small and might have low copy numbers, which probably increases the difficulty to meet sequencing requirements. Considering that neither all *R. solanacearum* strains have plasmid nor all sequenced plasmids share a synteny, the small plasmids might be dispensable for this strain, but the genes present in each plasmid may still have crucial functions. According to our results, the novel RipBS effector in the small plasmid may limit the host range of HA4-1 and contribute to or recede the pathogenicity in certain plant species. To explore the role of the plasmid in a bacteria, a plasmid-cured derivative is necessary, which allows a direct comparison between the plasmid-containing and plasmid-cured strains. For the stable plasmid, many curing agents and procedures are available for plasmid-cure (Trevors, 1986). However, there is no related research about the small plasmid of *R. solanacearum* strains at all. In the future, in-depth research can be carried out benefit from these small plasmids that have been sequenced. The small plasmid in HA4-1 can enrich the information of the plasmids in the *R. solanacearum* complex species and advance the correlational studies.

## Data Availability

The whole genome sequence of *R. solanacearum* HA4-1 has been deposited in GeneBank under the accession numbers CP022481 (chromosome), CP022482 (mega-plasmid), and CP022483 (plasmid). The strain HA4-1 is accessible in the Key Laboratory of Potato Biology and Biotechnology, Ministry of Agriculture and Rural Affairs, Huazhong Agricultural University, Wuhan, China.

## Author Contributions

XT, FL, and HC designed the experiments. XT performed the experiments and wrote the manuscript. HQ, MH, and WL helped to perform the PCR. DC, BW, YL, and KS contributed to the inoculation assay. BS, XZ, and CX helped to conceive the manuscript. HC and JD revised the manuscript.

## Conflict of Interest Statement

The authors declare that the research was conducted in the absence of any commercial or financial relationships that could be construed as a potential conflict of interest.

## Footnotes

1 https://www.ncbi.nlm.nih.gov/genome/genomes/490

2 https://iant.toulouse.inra.fr/bacteria/annotation/site/prj/T3Ev3/

3 http://sequence.toulouse.inra.fr/R.solanacearum

4 https://www.ncbi.nlm.nih.gov/genome/490?genome_assembly_id=360769
