# Supplementary material for: Complete Genome Sequence of Sequevar 14M Ralstonia solanacearum Strain HA4-1 Reveals Novel Type III Effectors Acquired Through Horizontal Gene Transfer
Source: Front Microbiol. 2019 Aug 14;10:1893. doi: 10.3389/fmicb.2019.01893 (PMC6703095; doi:10.3389/fmicb.2019.01893)
Supplement: Supplementary file 1 [file Presentation_1.pptx]

## Slide 1
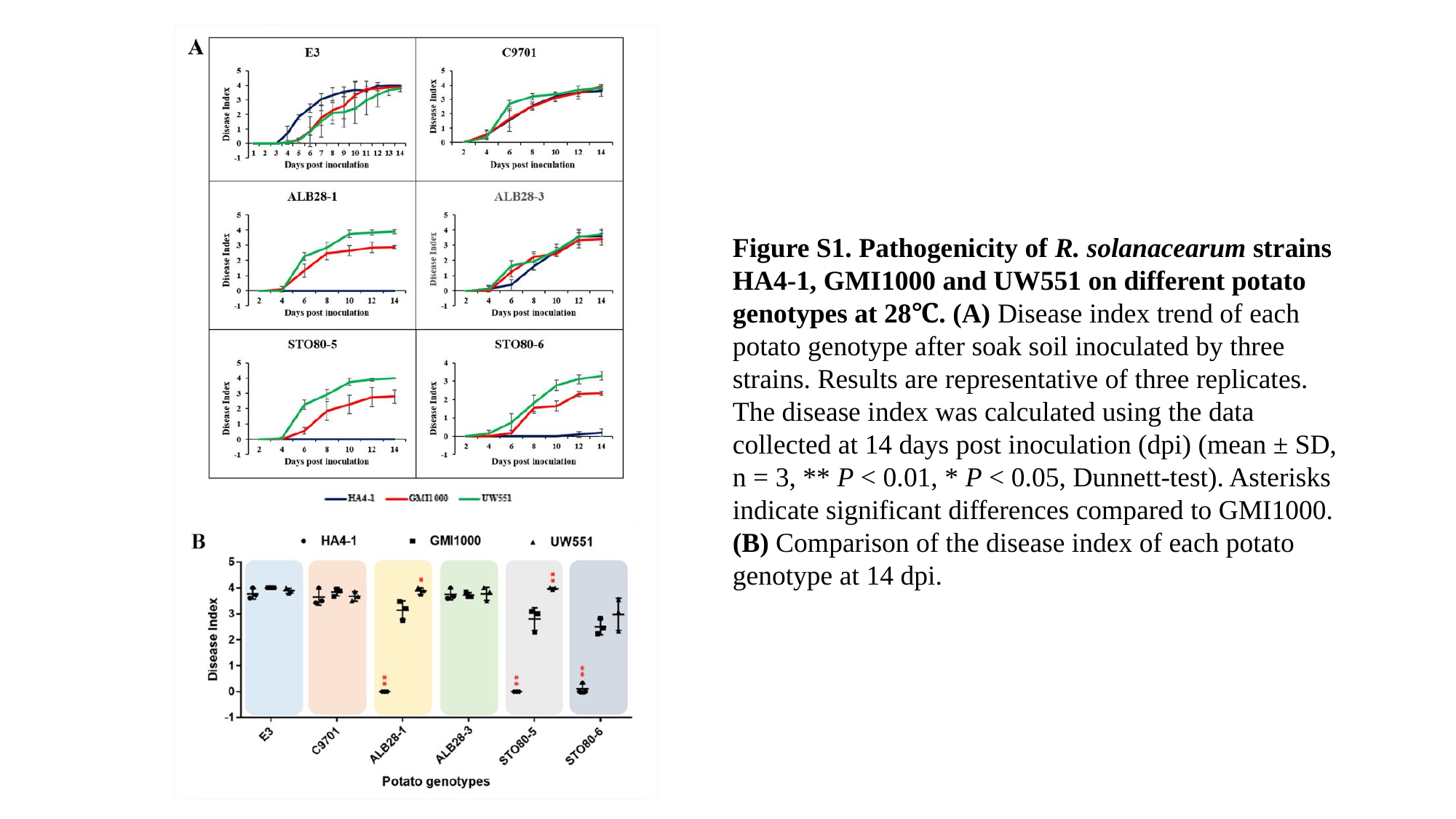

Figure S1. Pathogenicity of R. solanacearum strains HA4-1, GMI1000 and UW551 on different potato genotypes at 28℃. (A) Disease index trend of each potato genotype after soak soil inoculated by three strains. Results are representative of three replicates. The disease index was calculated using the data collected at 14 days post inoculation (dpi) (mean ± SD, n = 3, ** P < 0.01, * P < 0.05, Dunnett-test). Asterisks indicate significant differences compared to GMI1000. (B) Comparison of the disease index of each potato genotype at 14 dpi.

## Slide 2
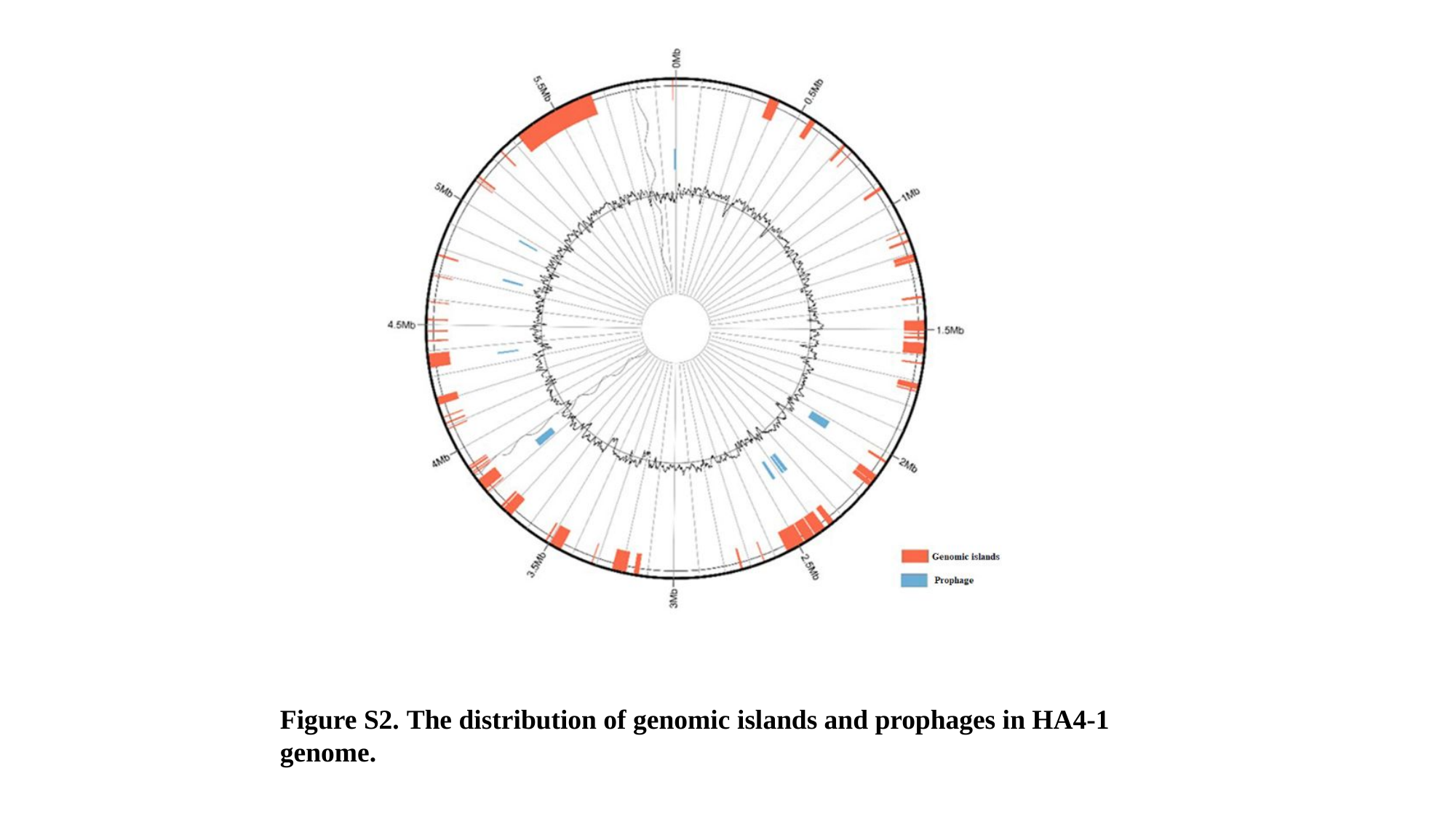

Figure S2. The distribution of genomic islands and prophages in HA4-1 genome.

## Slide 3
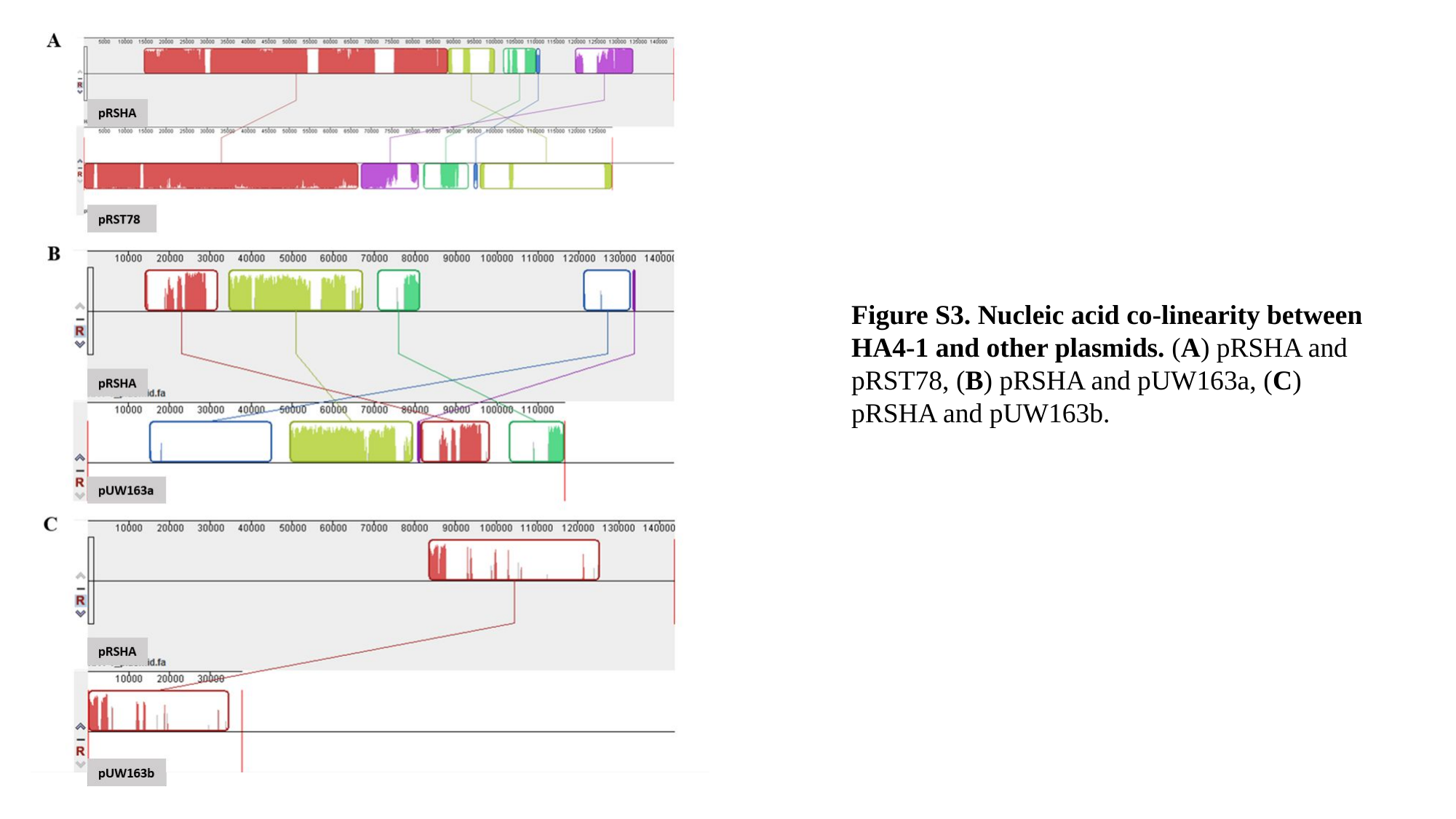

Figure S3. Nucleic acid co-linearity between HA4-1 and other plasmids. (A) pRSHA and pRST78, (B) pRSHA and pUW163a, (C) pRSHA and pUW163b.

## Slide 4
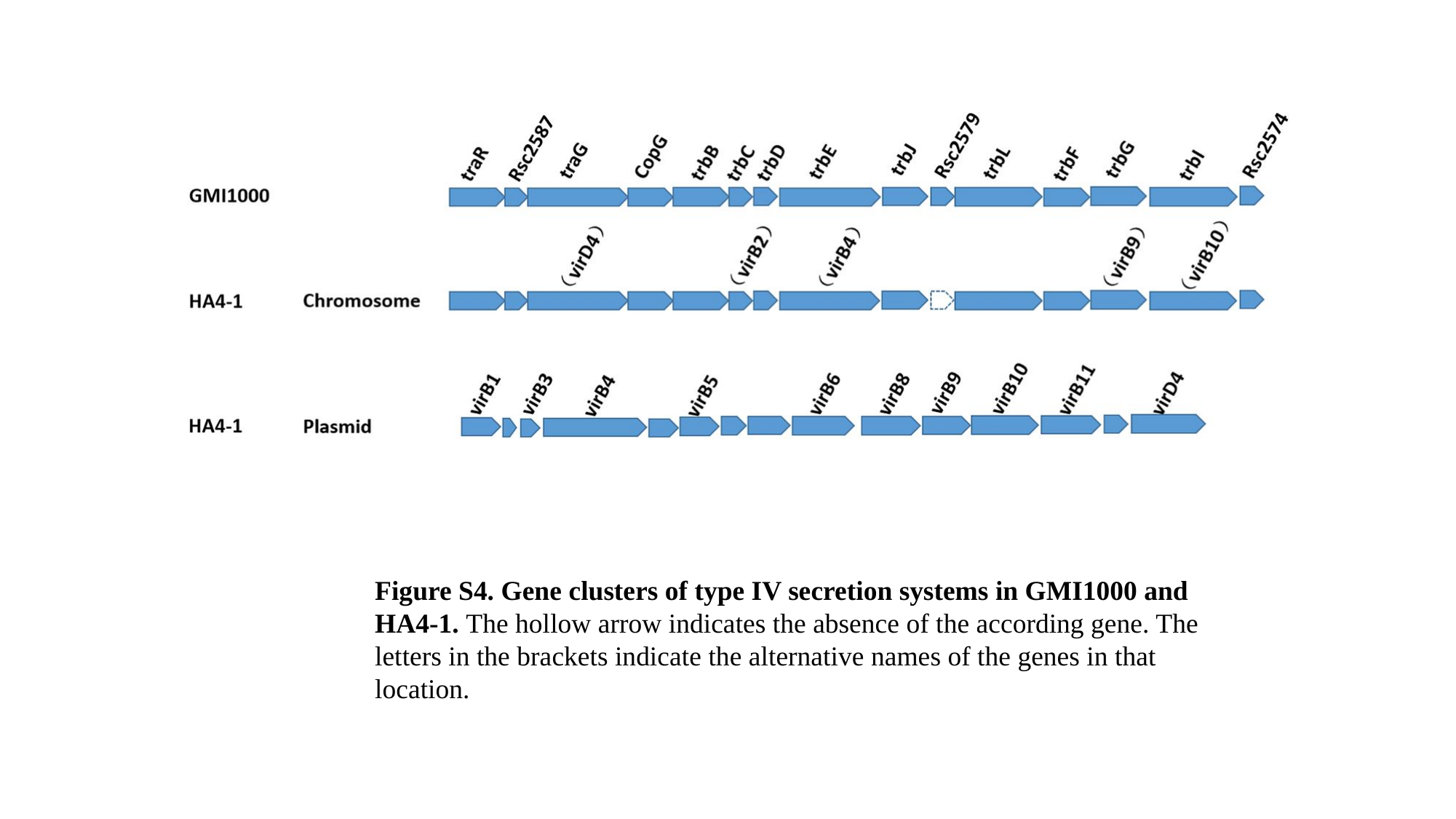

Figure S4. Gene clusters of type IV secretion systems in GMI1000 and HA4-1. The hollow arrow indicates the absence of the according gene. The letters in the brackets indicate the alternative names of the genes in that location.

## Slide 5
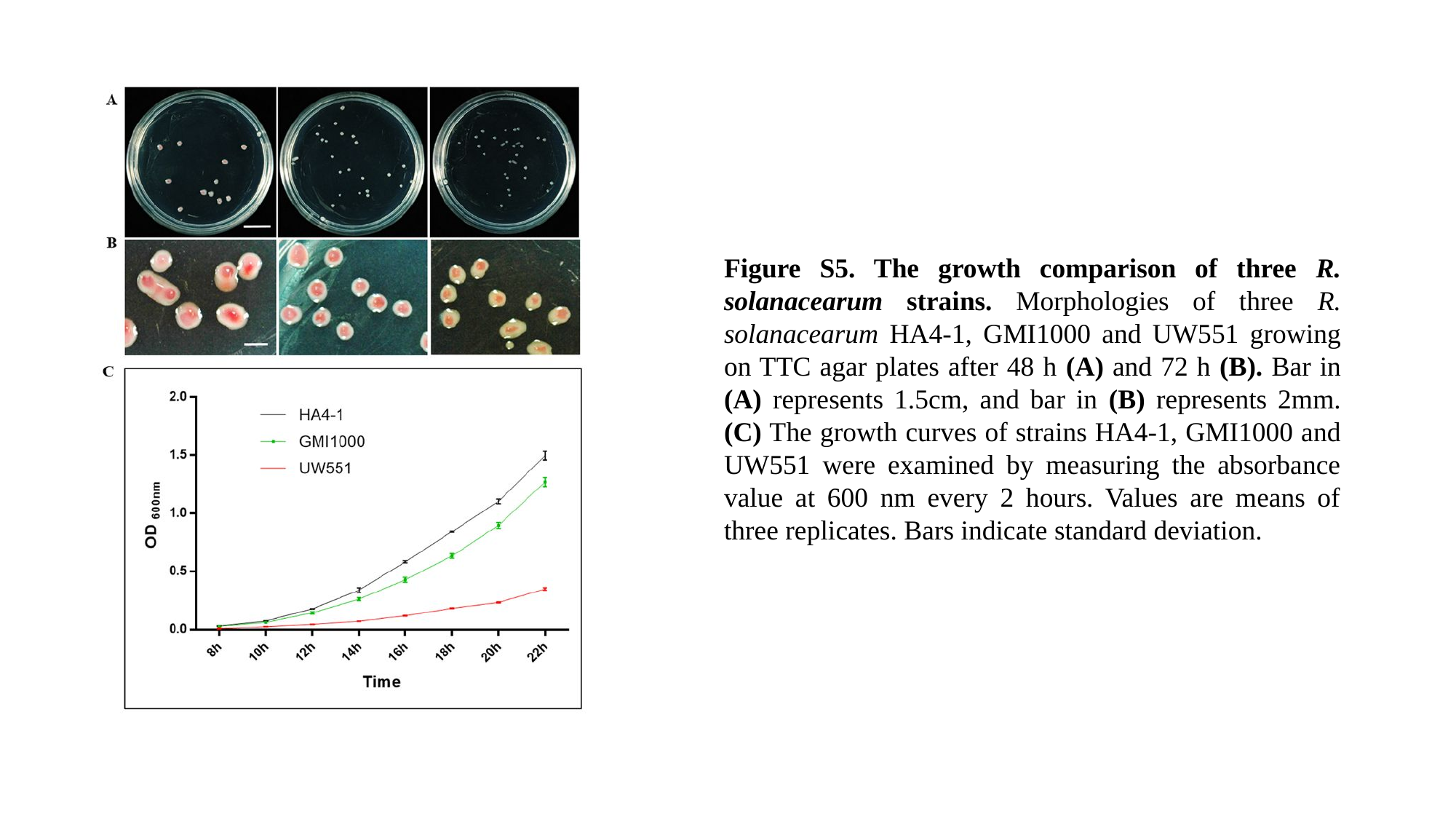

Figure S5. The growth comparison of three R. solanacearum strains. Morphologies of three R. solanacearum HA4-1, GMI1000 and UW551 growing on TTC agar plates after 48 h (A) and 72 h (B). Bar in (A) represents 1.5cm, and bar in (B) represents 2mm. (C) The growth curves of strains HA4-1, GMI1000 and UW551 were examined by measuring the absorbance value at 600 nm every 2 hours. Values are means of three replicates. Bars indicate standard deviation.

## Slide 6
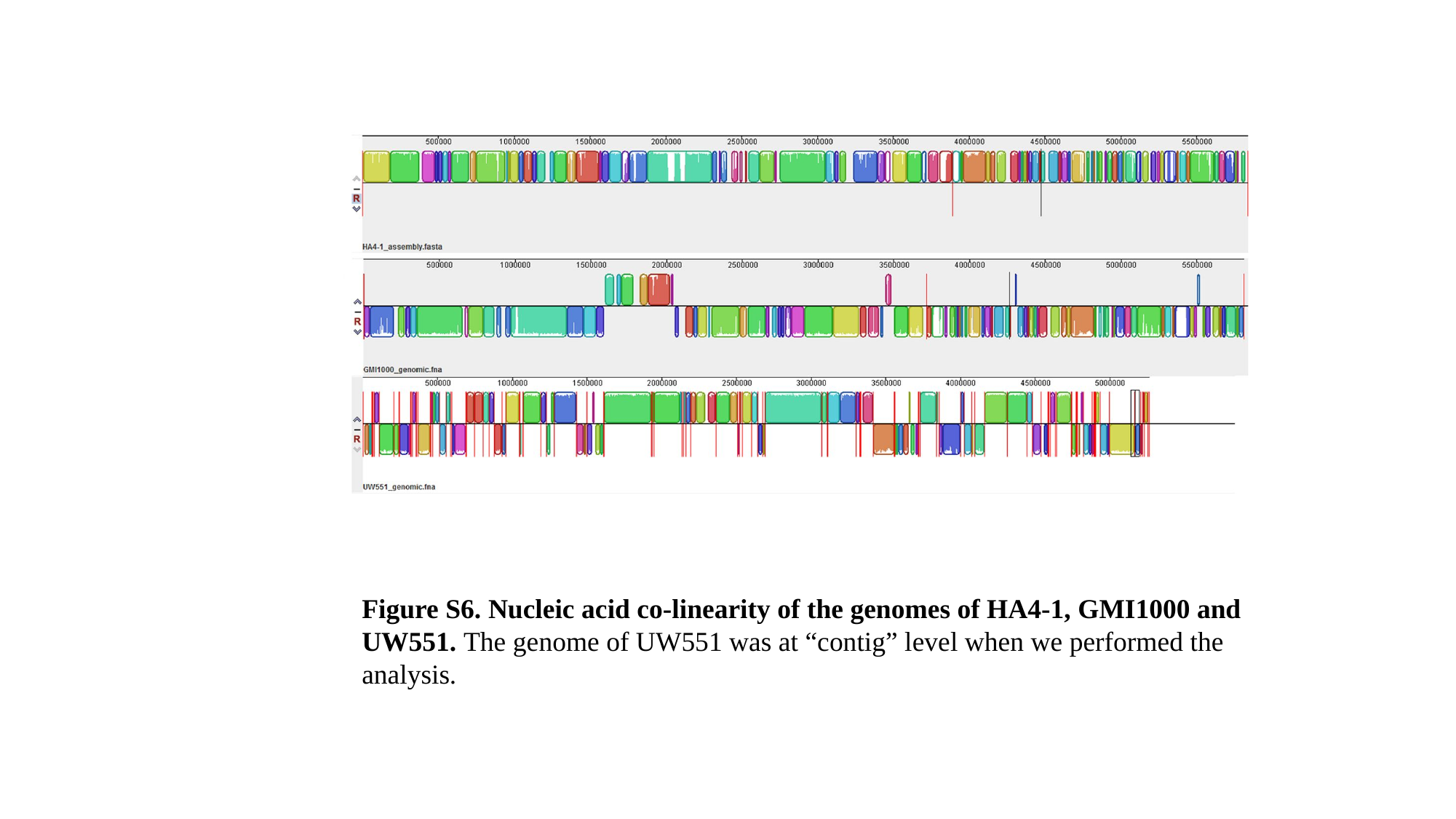

Figure S6. Nucleic acid co-linearity of the genomes of HA4-1, GMI1000 and UW551. The genome of UW551 was at “contig” level when we performed the analysis.
