# Supplementary material for: Complete Genome Sequence of Sequevar 14M Ralstonia solanacearum Strain HA4-1 Reveals Novel Type III Effectors Acquired Through Horizontal Gene Transfer
Source: Front Microbiol. 2019 Aug 14;10:1893. doi: 10.3389/fmicb.2019.01893 (PMC6703095; doi:10.3389/fmicb.2019.01893)
Supplement: Supplementary file 2 [file Table_1.DOCX]

**Table S1.Potato genotypes and and *R. solanacearum* strains used in this study.**

| **Material number** | **Species** |
| --- | --- |
| E3 | *Solanum tuberosum* |
| C9701 | *Solanum chacoense* |
| ALB28-1 | *Solanum albicans* |
| ALB28-3 | *Solanum albicans* |
| STO80-5 | *Solanum stoloniferum* |
| STO80-6 | *Solanum stoloniferum* |
| **Strains** | **Phylotype/Sequevar** |
| HA4-1 | I/14M |
| GMI1000 | I/18 |
| UW551 | IIB/1 |
