# Supplementary material for: Complete Genome Sequence of Sequevar 14M Ralstonia solanacearum Strain HA4-1 Reveals Novel Type III Effectors Acquired Through Horizontal Gene Transfer
Source: Front Microbiol. 2019 Aug 14;10:1893. doi: 10.3389/fmicb.2019.01893 (PMC6703095; doi:10.3389/fmicb.2019.01893)
Supplement: Supplementary file 3 [file Table_2.DOCX]

| **Location** | **GI Number** | **Total Size** | **P-Size^†^** | **Gene Number** | **P-Gene^‡^** |
| --- | --- | --- | --- | --- | --- |
| Chromosome | 36 | 793,355 | 20% | 777 | 23% |
| Mega-plasmid | 19 | 500,131 | 26% | 215 | 14% |
| Plasmid | 1 | 4,460 | 3% | 5 | 4% |
| Total | 56 | 1,297,946 | 49% | 997 | 41% |

**Table S2.** **General features of genomic islands in HA4-1 genome.**

†, the percentage of the GI region size.

‡, the percentage of the GI related genes.
