# Supplementary material for: Complete Genome Sequence of Sequevar 14M Ralstonia solanacearum Strain HA4-1 Reveals Novel Type III Effectors Acquired Through Horizontal Gene Transfer
Source: Front Microbiol. 2019 Aug 14;10:1893. doi: 10.3389/fmicb.2019.01893 (PMC6703095; doi:10.3389/fmicb.2019.01893)
Supplement: Supplementary file 4 [file Table_3.DOCX]

| **Location** | **Region number** | **Total size (bp)** | **P-Size^†^** | **Prophage number** | **Gene number** | **P-gene^‡^** |
| --- | --- | --- | --- | --- | --- | --- |
| Chromosome | 5 | 131822 | 3% | 148 | 141 | 4% |
| Mega-plasmid | 3 | 28334 | 1% | 31 | 27 | 2% |
| Plasmid | 1 | 9601 | 7% | 9 | 9 | 7% |
| Total | 9 | 169757 | 12% | 188 | 177 | 13% |

**Table S3.** **General features of prophages in HA4-1 genome.**

†, the percentage of the prophages region size.

‡, the percentage of the prophage related genes.
