# Supplementary material for: Complete Genome Sequence of Sequevar 14M Ralstonia solanacearum Strain HA4-1 Reveals Novel Type III Effectors Acquired Through Horizontal Gene Transfer
Source: Front Microbiol. 2019 Aug 14;10:1893. doi: 10.3389/fmicb.2019.01893 (PMC6703095; doi:10.3389/fmicb.2019.01893)
Supplement: Supplementary file 5 [file Table_4.DOCX]

| **Strain** | **Phylotype** | **Plasmid name** | **Plasmid Size(bp)** | **Reference** |
| --- | --- | --- | --- | --- |
| HA4-1 | I | pRSHA | 143,755 | This study |
| T78 | I | pRST78 | 128,742 | Cho et al.,2019 |
| UW163 | II | pUW163a | 116,523 | Ailloud et al., 2015 |
|  |  | pUW163b | 37,782 |  |
| CMR15 | III | pRSC35 | 35,008 | Remenant et al., 2010 |
| PSI07 | IV | pRSI13 | 12,811 | Remenantet al., 2010 |
| M4S | ND | pJTPS1 | 6,633 | Shimizu et al., 2000^†^ |

**Table S4.** Sequenced small plasmids in R. solanacearum strains.

†, sequences were direct submitted in NCBI.
